# The presence of wild edible plants and determinants influencing their harvest, consumption, and conservation in south eastern Bhutan

**DOI:** 10.1371/journal.pone.0285936

**Published:** 2023-10-10

**Authors:** Ram Chandra Bajgai, Yadunath Bajgai, Stephen B. Johnson

**Affiliations:** 1 Department of Environment & Life Sciences, Sherubtse College, Royal University of Bhutan, Kanglung, Tashigang, Bhutan; 2 National Potato Program, National Centre for Organic Agriculture, Yusipang, Thimphu, Bhutan; 3 Department of Agriculture, Ministry of Agriculture and Forests, Thimphu, Bhutan; 4 Department of Primary Industries, Weed Research Unit, Invasive Species Biosecurity, Adelaide, New South Wales, Australia; Washington University, UNITED STATES

## Abstract

**Definition:**

Wild edible plants (WEPs) grow naturally in self-maintaining ecosystems. WEPs are harvested for consumption, sale, and medicinal uses. We hypothesize that WEPs play a major role in supplying food and generating income for the rural people in a world that is increasingly recognising its emerging conservation issues. We tested this hypothesis by identifying the reasons for harvest, consumption, and conservation of WEPs using focus group discussion, field observations and questionnaire surveys in south eastern Bhutan in late 2019.

**Methods:**

Focused group discussions were held with the local people to identify reasons for harvest and consumption of WEPs. Data on the identified reasons for harvest, consumption, and conserving WEPs were determined using a questionnaire survey with ranking scales for a set of 76 randomly selected households. Representative field-observations and questionnaire surveys were carried out in villages close to forests. Parts of the plant used, how these were consumed, harvest season, and plant (life form) were recorded. The data was subjected to a Kruskal-Wallis rank sum test and weighted averages calculated.

**Result and conclusion:**

A total of 120 WEPs belonging to 63 families (including Agaricaceae) were reported. Most of the WEPs recorded were trees (45.0%) then herbs (25.8%), vines (13.3%) and shrubs (10.8%). The commonly consumed plant parts were the fruit (43.3%), shoots (28.3%) and leaves (20.8%).

The purposes for harvesting and consumption, conservation of WEPs were significantly (P<0.001) different, while the motivations for collecting WEPs were not. The motivation for collecting WEPs were family consumption > sale > medicinal uses > preservation for future use > insufficient food from cultivated source’s. The two most important strategies for conservation were to domesticate the WEPs and cultivate in forests. The findings reveal valuable lessons and insights about the reasons for harvesting, collection, consumption, and conservation of WEPs.

## Introduction

### Background

Forests represent a crucial resource stock for local people [1]. They supply a diverse array of foods, vegetables, medicines, firewood, timber, handicraft materials, etc. to communities [2,3]. Wild Edible Plants (WEPs) are plant species that are harvested for human consumption: they are critical for inclusive world food security [4]. Wild Edible Plants are defined as plants which grow naturally among self-maintaining populations in natural or semi-natural ecosystems and can exist independent of direct human action [5,6].

Humans have been using forests intensively since the dawn of civilization and this has often resulted in the wise management of forest resources [6]. These people harvest WEPs for self-consumption, and to meet supply of an array of livelihood commodities [7–9]. WEPs provide important nutrients and bioactive compounds in traditional diets which offer valuable health benefits [10]. There is a positive correlation between poverty and reliance on Non-Timber Forest Products [11] and WEP consumption and food security of local communities [12]. There is also a positive correlation between humans and their genes with diets derived from the environment; some claim that contemporary diets and lifestyles devoid of these diets underlie many of the chronic health problems of humans today [13]. Further, human malnutrition often coexists with lifestyle diseases such as overweight/obesity [14] often due to these socio-economic-environmental changes. Changes in traditional landscapes, cultures, and food chains compel communities towards adverse nutritional transitions affecting human health negatively [15]. In addition, consumption of highly-processed foods negatively affects human health and leads to undesirable outcomes such as gut inflammation [16].

### WEPs have food, nutritional and medicinal value

Approximately two billion people globally experience hunger due to lack of nutrition [17] and WEPs have the potential to help address the food security [18]. Numerous WEP species in any particular area are traditionally used as vegetables to supply nutrition and boost food security of the local communities [18,19]. WEPs are cheap and highly suitable alternative sources of healthy and nutritious foods and are vitally important for supporting the global food basket in general, particularly in sub-Saharan Africa [20]. In the face of changing environments, and food dynamics due to market chain disruptions and globalization, current food systems are waning in their ability to deliver a healthy diet [21]. Age-old traditional diets are recognized in offering prized health benefits [10] because WEPs form vital dietary sources to the rural poor [22,23]. Moreover, dishes prepared from WEPs have become special delicacies even in the menu of urbanites [24]. Dependency on WEPs for the supply of food is still common among rural communities of developing [25] and developed countries [26].

Many indigenous populations have relied on wild products for aeons in order to eat a balanced diet particularly during times of critical food shortages [9,27,28]. In their review of literature, Pinela et al [29] highlighted the importance of the role of WEPs in supplying important nutrients. Some WEPs contain a minimum daily intake amounts of essential nutrients [30]. Traditional foods are richer in micronutrients than conventional cultivated foods [31], and perfectly complement a diet supplied by conventionally cultivated crops. WEPs supply bioactive compounds/functional foods which can contribute to healthy diets that boost immunity helping to protect against an array of illnesses [30,32]. A correlational study on WEPs and diets conducted in a traditional community in Indonesia found that anaemic conditions among pregnant women are associated with a deficiency of vegetables and fruits in daily diets sourced from conventionally cultivated crops [33]. The abundance of many WEPs could help improve this situation [34]. WEP foods present a viable alternative option for alleviating micronutrient deficiency among rural and indigenous people [35]. Further, in a comparative study, WEP consumers had a significantly higher intake of Vitamins A, B6, and C, and calcium [36]. Despite the huge potential of WEPs to supply myriad of nutrients to the consumers, WEPs are not recognized as a noteworthy contributor to the food basket, particularly in the developed countries [37]. Besides dietary nutrients, many WEPs also have medicinal properties [38,39]. Diverse wild plants consumed in India are also reported to have a range of medicinal uses [3]. In Bhutan, this bi-dimensional use of WEPs is commonly reported, for example by Wangyal [40], Zangpo, Tshering [41], and Dema and Dolkar [42]. A study in Tibetan plateau reported the use of thirty-one WEP species as medicines to treat gastropathy, coughs, fevers, rheumatism, dysentery, fractures, dyspepsia, hemoptysis, and asthma, in addition to their use as special tonics [43].

### WEPs have traditional and economic value

Traditional knowledge of WEPs is associated with indigenous food systems [44]. Culinary preparations of certain WEPs are often delicacies and are consumed during major festivals [3]. Similarly, WEPs are associated with local festivals, rituals and funerals in Khoma, a village in the Lhuentse district in Bhutan [42]. Likewise, varieties of wild *Dioscorea* species are harvested and consumed in special preparations during Losar (New Year), *Lochoe* (an annual ritual) and *Prechula* (an offering to local deities) in the Kheng region of central Bhutan [28]. Valuable indigenous knowledge on the preparation and use of many traditional dishes is being lost over time, triggered by rural to urban lifestyle changes [45].

The decline in the availability of WEPs also diminishes the valuable traditional knowledge pertaining to diverse use of WEPs [36]. A study from Khaling in the Trashigang district in eastern Bhutan on forest ecosystem services availed by the locals showed that Nu. 21.6 million (1 USD ≈ Nu.76) worth of forest commodities are produced annually [46].

There is also a vast and valuable traditional knowledge that Bhutanese farmers have not documented around traditional health care systems in Bhutan [47], on forest resource management [40], soil fertility management [48], and on different aspects of WEPs [25,28,40,41,46,47,49–60]. Since food colonialism erodes local food systems, it is now time for the opposite, a valorization of indigenous food systems [61].

### The conservation of WEPs

The potential loss of some WEPs is of global conservation concern. Species has been for example *Psilotrichum axilliflorum* is listed as endangered, and *Mitragyna stipulosa* and *Vitellaria paradoxa* are listed as vulnerable [39]. Unregulated and over-exploitation of forest resources results in disharmony between forest resource consumers [50]. Declines in species density and biodiversity richness in the natural habitat occur [24,62].

**Fig 1:**
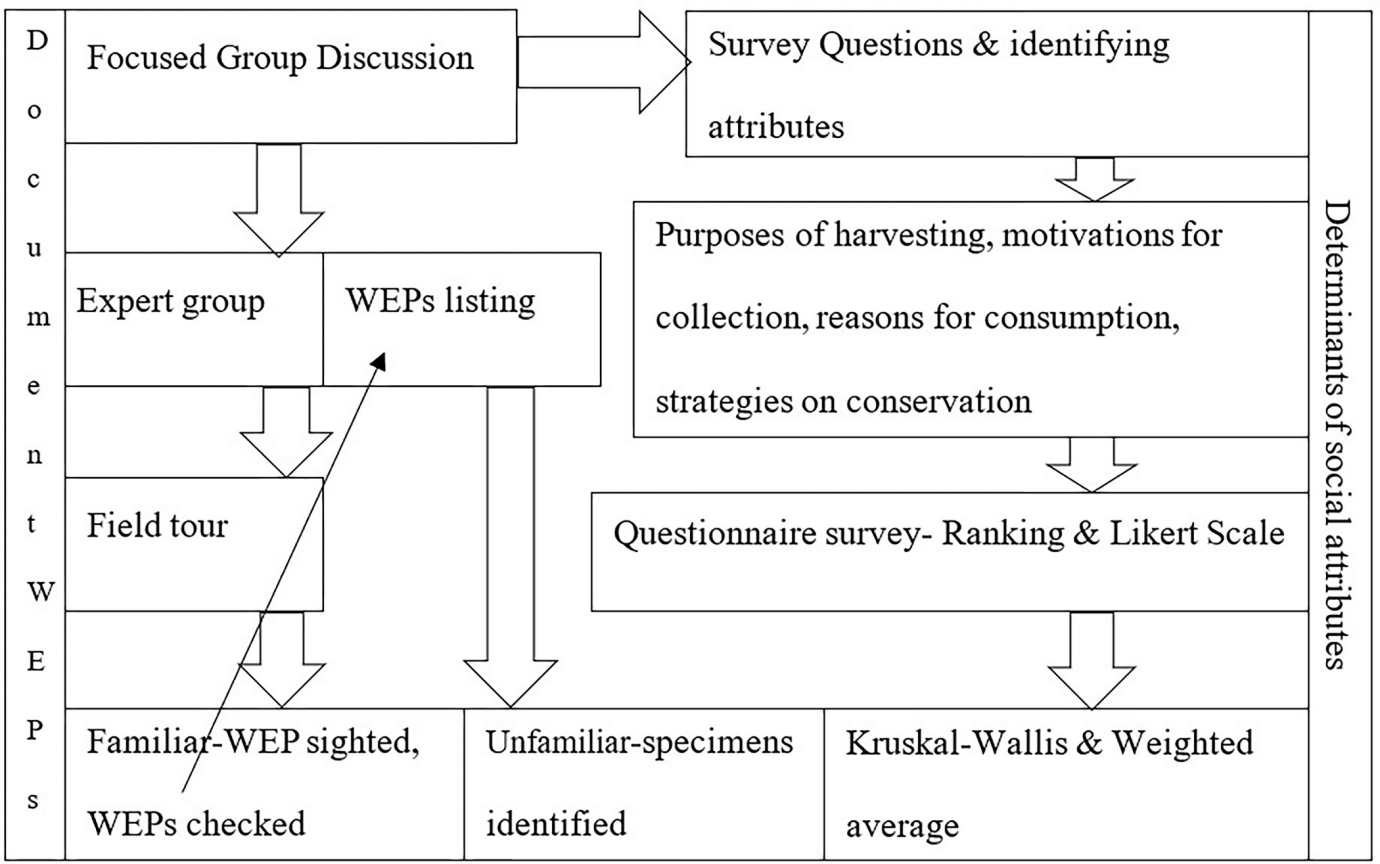
Schema of conceptual framework of the study.

There is a widespread ignorance about the loss of WEPs diversity and decline in abundance at policy level [e.g. 63,64]. A recent review by Bajgai [50] showed a lack of clear quantification of the food and nutritional benefits supplied by WEPs: this resulted in the potential benefits of WEPs being overlooked and omitted from mainstream developmental programs [6,19]. These omissions could have been driven by a compulsion to produce cash crops and starchy staples [65] that have featured strongly in governmental development agenda. Additionally, a severe lack of nutrient composition data results in limited quantification of the benefits of WEP, and their dietary contribution [35,50]. In contrast, unrestricted access to WEPs may have resulted in oversupply and a lowering of commercial value in the past, further reducing WEP prominence image [66]. Since it is quite unrealistic to single out any particular harvesting method as sustainable [67], developing agro-techniques for the mass production of WEPs for conservation and for meeting the nutritional and livelihood needs of indigenous communities [3,5,19]. Alarmingly, WEPs are under constant threat from habitat destruction from anthropogenic (livestock grazing, agricultural land expansion and over-exploitation of WEPs) and natural phenomena (forest fire, and climate change), which collectively cause indirect loss of valuable traditional knowledge in Bhutan [42]. Despite this, there is a general lack of understanding about the role WEPs have in food system and the socio-economy of Bhutanese. We hypothesize that WEPs play a major role in supplying food and generating income for rural people in the face of emerging conservation issues. We tested this hypothesis to identify the purposes for harvesting, consumption, and conservation of WEPs using focus group discussion and field surveys in the villages close to forests in south eastern Bhutan. The study also aims to determine the presence and uses of WEPs species in the study area. A simplified conceptual framework of the study is presented to depict the major steps involved (Fig 1).

## Methodology

### Study area

Bhutan lies between the coordinates 26.7263 and 28.2466° N, and 88.7495 and 92.1236° E in the eastern Himalayas. It is geographically located between India in the East, West and South, and China in the North. Bhutan is divided into twenty districts. Samdrup Choeling is one of the sub-districts (*dungkhag)* of Samdrup Jongkhar district and lies between the coordinates of 26.7848 and 27.2473° N and 90.9981 and 92.1187° E in south eastern Bhutan. Samdrup Choeling *dungkhag* is further divided into four administrative areas *viz*. Samrang, Pemathang, Phuntshothang and Martshala. Samdrup Choeling *dungkhag* is located 68 km away from the district headquarters of Samdrup Jongkhar. A modern motorable road connects it. Similarly, modern bitumen roads connect each administrative area; and farm roads (unsealed) connect the villages within. There are also government enterprises such as coal mines, fisheries, poultry farming and dairy farming on small and medium scales, and public institutions like schools (primary, lower secondary, middle secondary and higher secondary), health care and Renewable Natural Resources (RNR) centres. Apart from the local inhabitants, there are also public servants; private employees and business people living in the *dungkhag*. Most importantly, there is a designated area for weekend’s market at Tshangchutham village (Phuntshothang). The present study was conducted in the nearby forests surrounding the villages (Khateythang, Mindrupling, Khamethang, Tshangchum, Pemathang, Dungkharling, Chichhosa, Drupchhugang, Zhomlatthang and Majwa) of Samrang, Pemathang, Phuntshothang and Martshala areas. Samdrup Choeling is surrounded by evergreen tropical forest covering the foothills, plateaus, and plain lands. The sub-district has subtropical climatic conditions with annual rainfall of 3582 mm, average monthly minimum temperature of 16.72°C and average monthly maximum temperature of 25.94°C [68]. Predominantly agrarian, farmers in the study area practice subsistence agriculture.

### Qualitative method-focused group discussion

A village nearby a forest was purposively selected in each of the four areas (Geogs) of Samrang, Pemathang, Phuntshothang and Martshala under Samdrup Choeling sub-district where focus group discussions were held in November and December 2019. Members of the focused groups were selected in consultation with heads of villages (*Tshogpa)* and consisted of those who were aware of the use and knowledgeable of WEPs. Eight to 10 village elderlies of both the genders represented the group in a one-day consultation meeting in the participants’ mother tongue to discuss the availability and uses of WEPs in each village. The identified WEPs along with the characteristics of each WEP were recorded. Additionally, the focus group discussions helped formulate the questions for the follow-up questionnaire survey with particular emphasis on identifying the reasons for (a) harvest, (b) collection, (c) consumption, and (d) conservation of WEPs. Five representative attributes or ranks for each of these four major reasons were selected though group discussions and consensus. At the end of each focus group discussion, a key informant was identified to assist in constructing a sampling frame or system for the field survey.

### Field survey

Following the focus group discussions, a semi-structured questionnaire field survey was conducted in the Samdrup Choeling sub-district to assess the factors that influence the use and conservation of WEPs. The surveyors were trained prior to the survey to ensure uniformity in execution of the survey conducted between January and October 2020. The identified key informant helped with the sampling frame of the relevant households prior to the implementation of the questionnaire survey. Purposive sampling was used because this method allowed random selection of households without biases [69]. A total of 76 randomly selected farmers from the sampling frame were interviewed, which was more than the required 5% sample size recommended for a representative study [70]. Prior to an interview, each farmer was briefed and asked for consent and only farmers who consented were included in the focused group discussion and responses to questionnaires. Each set of five questions (attributes/ranks) on the four key aspects of purposes of harvesting, motivations for collection, reasons for consumption, and strategies on conservation of WEPs identified in the focus group discussion were covered in the questionnaire survey. The five attributes/ranks under each of the four major aspects were written in the question response format and were ranked during the interview based on the respondents’ priority on a 5-point scale (1 = lowest priority, 2 = low priority, 3 = moderate priority, 4 = high priority and 5 = highest priority) to quantitate the qualitative responses for statistical analysis [71,72]. Such scoring is a simple and flexible way of collecting data for a farmer perception study [73,74].

The WEPs identified and listed during focused group discussion were assessed in the wild and in the cultivated/fallow land to ascertain the presence in the sub-district. In other words, a species presence verification was conducted along with the questionnaire survey. Herbarium specimens of unfamiliar plants were collected, later identified using The Flora of Bhutan [75] and other literature [2,76–78], and stored in the Life Science Museum of Sherubtse College, Royal University of Bhutan, Trashigang. Other key information on plant parts used, season of harvest, type of plant (life form), and consumption was also recorded. Post-field survey focused group discussions were held to discuss usage and consumption. Respondents’ demographic indicators of gender, age range and literacy level were also captured. The minimum age of a respondent was set at 20 years to ensure that the responses were more reliable.

### Statistical analyses

Since the data generated in this study were non-parametric in nature (and not fulfilling the assumptions of an ANOVA), a Kruskal-Wallis test by ranks (equivalent to one-way ANOVA for parametric data) were used for statistical data analyses using the IBM SPSS Statistics (Version 22) predictive analytics software. This methodology is more appropriate to analyse non-parametric or ordinal data [79,80]. The data collected on the five attributes/ranks under the major aspects of (a) purposes of harvesting, (b) motivation for collection, (c) reasons for consumption, and (d) strategies on conservation of WEPs were assessed using a boxplot and then subjected to Kruskal-Wallis test by ranks. Significantly, different values were segregated using a Dunn test to make inferences across the attributes. Raw data were processed and computed using Microsoft Excel. Plots were generated using Microsoft Excel and the Statistical Tool for Agriculture Research (STAR version 2.0.1). To substantiate and compare with the inferential statistical analysis, weighted averages were calculated. The responses of the farmers’ rankings were organized in descending order and compared. For this, a weighted average, with weights of 1 to 5 were assigned corresponding to the ranking scale of 1 to 5, and then calculated using a simple arithmetic formula (Eq 1)

$$X=\frac{\sum_{i=1}^{n}w_{i}x_{i}}{\sum_{i=1}^{n}w_{i}}$$ (1)

where, X is the weighted average mean, x_i_ is the multiset of data, and w_i_ is the weights (1–5) assigned to each of the data sets. The generated Kruskal-Wallis test by ranks were plotted along with the calculated weighted averages so that comparisons could be made.

## Results

### Presence, consumption, plant type and harvest season of WEPs

A total of 120 species from 63 families (including one Agaricaceae) of wild edible plants were reported in the study sites (Table 1). Most of the species are consumed cooked (46.7%), raw (39.2%), pickled (15.8%), chewed (8.3%), and in other forms (Table 2). In terms of plant parts, people consumed 43.3% species as fruits, shoots (28.3%), leaves (20.8%), flowers (10.8%), and seeds (6.7%), or as whole plant (4.2%). The reported WEPs were trees (45.0%), herbs (25.8%), vines (13.3%), shrubs (10.8%), perennial grasses (4.2%) and aquatic life forms (0.8%). Most of the WEPs were harvested in spring and summer (each 26.7%), followed by winter (18.3%) and autumn (16.9%): only 14.2% were harvested year-round. Over one-quarter of 120 species reported were considered to be invasive in nature (Table 1).

**Table 1:** Wild edible plants documented from the study sites and reported by focus groups of n = 8–10 expert elder participants from four areas- Samrang, Pemathang, Phuntshothang and Martshala in south eastern Bhutan. Plants are arranged alphabetically by local name.

Botanical name	Family	Local name	Type of plant	Season of harvest	Parts used	Method of consumption	Invasive*
*Lantana camara* L.	Verbenaceae	Aaputray	Shrub	All seasons	Ripened fruits and leaves	Ripened fruits eaten raw, leaves are chewed with the thick spines of *Bombax ceiba* as betel	Yes
*Adhatoda vasica* L.	Acanthaceae	Aasuro	Shrub	Spring	Flowers	Flower cooked as vegetable	Yes
*Rubus occidentalis* L.	Rosaceae	Aiselu	Shrub	Autumn	Fruits	Fruits eaten raw	Yes
*Fagopyrum dibotrys* (D.Don) H.Hara	Polygonaceae	Alujhar	Herb	Summer to autumn	Leaves and tender shoots	Whole plant when tender is used as vegetable	Yes
*Phyllanthus emblica* L.	Euphorbiaceae	Amala	Tree	Autumn to winter	Fruits	Fruits eaten raw and as pickled	No
*Spondias dulcis* L.	Anacardiaceae	Amaro	Tree	Spring to summer	Fruits, flowers, leaves and tender shoots	Ripened fruits, leaves and tender shoots eaten raw, flowers and tender fruits consumed after pickling	No
*Syzygium kurzii* (Duthie) Balak	Myrtaceae	Ambakay	Tree	Summer	Ripened fruits	Ripened fruits eaten raw	No
*Pteridium aquilinum* (L.) Kuhn	Dennstaedtiaceae	Aulay Niguru	Herb	All seasons	Tender shoot and leaves	Tender shoots and leaves eaten as vegetables	Yes
*Arisaema dracontium* (L.) Schott	Araceae	Baako	Herb	Summer to autumn	Young shoots	Young shoots cooked as curry	No
*Artocarpus lacucha* (Buch.-Ham.)	Moraceae	Badar	Tree	Spring	Ripened fruits	Ripened fruits eaten raw	No
*Chenopodium album* L.	Amaranthaceae	Baethu	Herb	Spring to summer	Leaves and young shoots	Leaves and young shoots cooked as vegetables	Yes
*Clerodendrum speciosissimum* Drapiez	Lamiaceae	Bakakane	Shrub	Spring	Young leaves	Shoot tips and young leaves cooked as vegetables	No
*Coccinia indica* (Wight & Arn)	Cucurbitaceae	Ban kakri	Vine	Summer	Fruits	Fruits eaten raw as well as cooked as curry	No
*Momordica dioica* (Roxb.)	Cucurbitaceae	Ban Karela	Vine	Summer to autumn	Fruits	Fruits cooked as vegetables	No
*Aglaia edulis* (Roxb.) Wallich	Meliaceae	Bandrey	Tree	Summer	Ripened fruits	Ripen fruits eaten raw	No
*Hovenia dulcis* Thunb.	Rhamnaceae	Bangi	Tree	Spring	Fruits (fleshy rachis)	Fleshy rachis eaten raw (ripened) and cooked	No
*Dioscorea belophylla* L.	Dioscoreaceae	Bantarul	Vine	Winter	Tubers, corm, tender shoots	Tubers and corm eaten like potato (boiled as well as curry), tender shoots/tips of vine cooked as vegetables	No
*Plectocomia himalayana* Griff.	Arecaceae	Bate	Shrub	All seasons	Tender stems	Tender stems cooked as vegetables	No
*Calamus flagellum* (Griff. ex Mart.)	Arecaceae	Bate (Phyakrey)	Vine	Autumn and spring	Fruits and tender stem	Fruit eaten like betel nut, and tender stem cooked as vegetable	No
*Costus speciosus* (J.Konig) C.Specht	Costaceae	Bate lauri	Herb	Autumn	Stem	Stem chewed for watery juice	No
*Ziziphus jujuba* Mill.	Rhamnaceae	Bayer	Tree	Winter	Ripened fruits	Ripened fruits eaten raw as well as pickled	Yes
*Terminalia bellerica* (Gaertn.) Roxb.	Combretaceae	Berro	Tree	Autumn to winter	Fruits	Fruits eaten raw	No
*Rhus glabra* L.	Anacardiaceae	Bhakmilo	Tree	Winter	Ripened fruits and seeds	Ripened fruit eaten raw, and seeds processed as pickle; it is also used for extraction of sour juice	No
*Girardinia diversifolia* (Link) Friis	Urticaceae	Bhangray sisnu	Herb	Summer	Leaves, tender shoots and flowers	Leaves, tender shoots and flowers eaten as vegetable	Yes
*Duchesnea indica* (Andrews) Th.Wolf	Rosaceae	Bhui Kafal	Herb	Winter	Ripened fruits	Ripened fruits eaten raw	No
*Boerhavia diffusa* L.	Nyctaginaceae	Bhui sag	Herb	Spring to autumn	Tender shoots and leaves	Tender shoots and leaves cooked as vegetables	Yes
*Dioscorea pentaphylla* L.	Dioscoreaceae	Bhyagur	Vine	Winter	Bulbils and tubers	Bulbils and tubers eaten like potato (boiled as well as curry)	No
*Bambusa clavata* Stapleton	Poaceae	Bijuli Baas	Perennial grass	Summer	Tender shoots	Tender shoots eaten as vegetable	No
*Citrus medica* L.	Rutaceae	Bimiro	Tree	Autumn to winter	Fruits	Fruits eaten raw as well as used for extraction of sour juice	No
*Paederia foetida* L.	Rubiaceae	Biri lahara	Vine	Spring to autumn	Tender shoot tips	Tender shoot tips cooked as vegetables	Yes
*Zanthoxylum rhetsa* (Roxb.) DC.	Rutaceae	Boketimur	Tree	Autumn	Fruits	Fruits are use as spice	No
*Oxalis corniculata* L.	Oxalidaceae	Chari amilo	Herb	Spring to autumn	Whole plant	Whole plant is eaten raw, in salads and pickled	Yes
*Diploknema butyracea* (Roxb.) H.J.Lam	Sapotaceae	Cheuri	Tree	Spring	Ripened fruits and seeds	Ripened fruits eaten raw, seeds used for extraction of butter	No
*Garcinia unitoria* (DC) Wt	Clusiaceae	Chhuyale	Tree	Autumn to winter	Fruits	Ripened fruits are eaten raw and used to extract sour juice	No
*Pentapanax fragrans* (D.Don) Ha	Araliaceae	Chindey	Tree	Spring	Tender shoots and leaves	Tender shoots and leaves eaten as vegetables	No
*Boehmeria* sp Jacq.	Urticaceae	Chiplay	Shrub	Spring	Tender shoots and leaves	Tender shoots and leaves cooked as vegetables	Yes
*Dendrocalamus hamiltonii* Gamble	Poaceae	Choya Baas	Perennial grass	Summer	Tender shoots	Tender shoots are pickled as well as cooked as vegetables	No
*Phlogacanthus thyrsiformis* (Roxb. ex Hardw.) Mabb.	Acanthaceae	Chuwa	Shrub	Spring	Flowers	Flowers eaten as vegetables	No
*Ardisia macrocarpa* Wall.	Primulaceae	Damaigeda	Shrub	Spring to summer	Ripened fruits	Ripened fruits eaten raw	No
*Boehmeria rugulosa* Weddell.	Urticaceae	Dar	Tree	All seasons	Gel extracted from the bark	Gel extracted from the bark is used as ingredient in Sel roti (kind of locally made rice dough nut)	No
*Ficus racemosa* L.	Moraceae	Dumri	Tree	All seasons	Ripened fruits and Resin	Ripen fruits eaten raw, resin that oozes from the bark is chewed as bubble gum	No
*Elatostema lineolatum* J.R.Forst. & G.Forst.	Urticaceae	Gaglato	Herb	All seasons	Young shoots	Young shoots cooked as vegetables	Yes
*Gynocardia odorata* R.Br	Achariaceae	Gantey	Tree	Summer	Seeds	Seeds used for extraction of vegetable oil	No
*Bridelia retusa* (L.) A.Juss.	Phyllanthaceae	Gayo	Tree	Winter	Dried fruits	Dried fruits eaten after roasting	No
*Hodgsonia macrocarpa* (Blume) Cogn.	Cucurbitaceae	Gheuphal	Vine	Winter	Ripened fruits	Ripened fruits eaten raw	No
*Achyranthes aspera* L.	Amaranthaceae	Ghoptey kada (Apamarga)	Herb	Spring to autumn	Tender shoots/leaves	Tender shoots/leaves cooked as vegetables	Yes
*Centella asiatica* (L.) Urban	Apiaceae	Ghortapray	Herb	Winter	Leaves	Leaves cooked as vegetables	Yes
*Dioscorea bulbifera* L.	Dioscoreaceae	Githa	Vine	Autumn to winter	Bulbils and tubers	Bulbils and tubers eaten like potato (boil as well as curry)	No
*Canarium strictum* Roxb.	Burseraceae	Gokul	Tree	Summer	Fruits	Fruits eaten raw	No
*Stephania glabra* (Roxb.) Miers	Menispermaceae	Gudargano	Vine	Spring to autumn	Tender shoot tips	Tender shoot tips cooked as vegetables	Yes
*Callicarpa arborea* Roxb.	Lamiaceae	Gueyeli	Tree	All seasons	Bark	Bark eaten for pleasure like betel nut	No
*Terminalia chebula* Retz.	Combretaceae	Harro	Tree	Autumn to winter	Fruits	Fruits eaten raw for medicinal purpose and soothing for taste	No
*Houttuynia cordata* Thunb.	Saururaceae	Hilay jhar	Herb	Summer to autumn	Root	Root is eaten as salad, as spice and vegetables	Yes
*Diplocyclos palmatus* (L.) C.Jeffrey	Cucurbitaceae	Indreni	Vine	Summer	Ripened fruits	Ripened fruits eaten raw	No
*Remusatia vivipara* (Roxb.) Schott	Araceae	Jaluko	Aquatic	Spring to autumn	Tender shoots	Tender shoots cooked as vegetables	Yes
*Syzygium cumini* (L.) Skeels	Myrtaceae	Jamuna	Tree	Winter	Ripened fruits	Ripened fruits eaten raw	No
*Mangifera sylvatica* Roxb.	Anacardiaceae	Jangali Aap	Tree	Summer	Fruits	Ripened fruits eaten raw and young fruits pickled	No
*Agaricus* sp. L.:Fr. emend Karst.	Agaricaceae	Jungali cheu	Herb	Summer	Fruiting body	Mushroom cooked as vegetable	No
*Eryngium foetidum* L.	Apiaceae	Jangali dhania	Herb	All seasons	Leaves	Leaves used as spice and condiment	No
*Artocarpus lanceifolius* Roxb.	Moraceae	Jangali katar	Tree	Summer	Fruits	Ripened fruits eaten raw and tender fruits cooked as vegetable and pickled	No
*Musa balbisiana* Colla	Musaceae	Jangali kola	Tree	All seasons	Flowers and ripened fruits	Flowers cooked as vegetables as well as pickled; and ripened fruits eaten raw	No
*Phytolacca acinosa* Roxb.	Phytolaccaceae	Jaringo	Herb	Spring	Tender shoots and leaves eaten	Tender shoots and leaves eaten as vegetables	No
*Capsicum annuum* (pequin)	Solanaceae	Jiray khursani	Shrub	Summer to autumn	Fruits, leaves and tender shoots	Fruits used as spice as well as pickle; tender leaves and shoots cooked as vegetables	No
*Ficus virens* Aiton	Moraceae	Kabro	Tree	Spring	Flowers	Flowers eaten as pickled	No
*Arundinaria* sp. Michx.	Poaceae	Kaday malingo	Perennial grass	Summer	Tender shoots	Tender shoots eaten as vegetables	No
*Solanum nigrum* L.	Solanaceae	Kali geda/Kanchi sag	Herb	Spring to autumn	Ripened fruits, leaves and tender shoots	Ripened fruits eaten raw; leaves and tender shoots are cooked as vegetables	Yes
*Bidens pilosa* L.	Asteraceae	Katarey Kuro	Herb	Spring to summer	Leaves and shoot tips	Leaves and shoot tips are consumed as tea after boiling	Yes
*Castanopsis indica* (Roxb. ex Lindl.) A.DC.	Fagaceae	Katus	Tree	Winter	Seeds	Seeds eaten raw as well as roasted	No
*Gmelina arborea* Roxb.	Lamiaceae	Khamari	Tree	Spring	Flowers	Flowers cooked as vegetables	No
*Ficus cunia* Buch.-Ham. ex Roxb.	Moraceae	Khaneu	Tree	Summer	Ripened fruits	Ripened fruits eaten raw	No
*Ficus semicordata* Buch.-Ham. ex Sm.	Moraceae	Khasrey Khaneu	Tree	Summer	Ripened fruits	Ripened fruits eaten raw	No
*Morus alba* L.	Moraceae	Kimbu (sanu)	Tree	Summer	Fruits	Fruits eaten raw	No
*Bauhinia variegata* L.	Fabaceae	Koiralo	Tree	Autumn	Flowers	Flowers cooked as vegetables	No
*Smilax orthoptera* A.DC.	Smilacaceae	Kukur daina	Vine	Summer	Tender shoot tips	Tender shoot tips cooked as vegetables	No
*Schleichera oleosa* (Lour.) Merr.	Sapindaceae	Kusum	Tree	Summer	Fruits	Fruits eaten raw	No
*Choerospondias axillaris* (Roxb.) B.L.Burtt & A.W.Hill	Sapindaceae	Lapsi	Tree	Autumn to winter	Fruits	Fruits eaten raw as well as pickled	No
*Amaranthus spinosus* L.	Amaranthaceae	Luday	Herb	Spring to autumn	Leaves and tender shoots	Leaves and tender shoots cooked as vegetables	Yes
*Ficus barteri* Sprague	Moraceae	Lute khaneu	Tree	Summer	Ripened fruits	Ripened fruits eaten raw	No
*Bambusa nutans* Wall. ex Munro	Poaceae	Maal Baas	Perennial grass	Summer	Tender shoots	Tender shoots pickled as well as cooked as vegetables	No
*Begonia malabarica* Lam.	Begoniaceae	Magar kajay	Herb	Autumn to spring	Tender plant	Tender plant cooked as vegetable	No
*Elaeagnus latifolia* L.	Elaeagnaceae	Maldido/ Madilo	Vine	Winter	Ripened fruits	Ripened fruits eaten raw as well as pickled	No
*Colocasia esculenta* (L.) Schott	Araceae	Manay	Herb	Winter	Corm and cormels	Corm and cormels cooked as vegetable	Yes
*Docynia indica* (Wall.) Decne.	Rosaceae	Mel	Tree	Autumn to winter	Fruits	Fruits eaten raw as well as pickled, and sweet-sour juice extracted	No
*Tupistra nutans* Wall. ex Lindl.	Asparagaceae	Nakima	Herb	Autumn	Flowers	Flowers cooked as vegetables	No
*Ficus auriculata* Lour.	Moraceae	Nibaro	Tree	All seasons	Ripened fruits	Ripened fruits eaten raw	No
*Diplazium esculentum* (Retz.) Sw.	Athyriaceae	Lekali Niguro	Herb	Spring to summer	Tender shoots	Tender shoots cooked as vegetable	Yes
*Juglans regia* L.	Juglandaceae	Okhar	Tree	Winter	Nut	Nutmeat is eaten raw	No
*Sterculia abbreviata* E.L.Taylor ex Mondragón	Malvaceae	Oodal	Tree	All seasons	Seed and resin	Nuts eaten roasted, resin from the bark added in Sel roti (locally made rice dough nut)	No
*Dillenia indica* L.	Dilleniaceae	Paachphalay	Tree	Autumn	Fruits	Fruits eaten raw, cooked as vegetable as well as pickle	No
*Piper betle* L.	Piperaceae	Paan	Vine	All seasons	Leaves	Leaves eaten with betel nut and lime	No
*Amaranthus hybridus* L.	Amaranthaceae	Palangay	Herb	Spring to autumn	Tender shoot and leaves	Tender shoot and leaves cooked as vegetables	No
*Entada rheedii* Spreng.	Fabaceae	Pangra	Vine	Winter	Nutshell	Nutshell is eaten for pleasure	No
*Nephrolepis cordifolia* (L.) C. Presl	Nephrolepidaceae	Paniamala	Herb	Winter	Round swollen root	Round swollen root tuber is eaten raw	No
*Physalis minima* L.	Solanaceae	Phakphakay	Herb	Winter	Ripened fruits	Ripened fruits eaten raw	Yes
*Crassocephalum crepidioides* (Benth.) S.Moore	Asteraceae	Phalphalay jhar	Herb	Spring to autumn	Tender shoots eaten as vegetable	Tender shoots eaten as salad	Yes
*Persea americana* Mill.	Lauraceae	Phuntshe/Gheuphal	Tree	Winter	Ripened fruits	Ripened fruits eaten raw	No
*Calamus erectus* Roxb	Arecaceae	Phyakrey	Shrub	Winter	Fruits	Fruits eaten as beetle nut	No
*Piper longum* L.	Piperaceae	Pipla	Vine	Summer	Fruits	Fruits are used as spice	No
*Prunus cerasoides* Buch.-Ham. ex D.Don	Rosaceae	Puyeu	Tree	Summer	Fruits	Fruit eaten raw	No
*Horsfieldia kingii* (Hook.f.) Warb.	Myristicaceae	Ram guwa	Tree	Winter	Fruits	Fruits eaten as betel for pleasure	No
*Caryota urens* L.	Arecaceae	Rangbang	Tree	All seasons	Pith and nut	Pith cooked as vegetable; nut eaten as betel for pleasure	No
*Cassia fistula* L.	Fabaceae	`Ratbirsey	Tree	Autumn	Fruit Jelly	Jelly from the ripened fruit is eaten	No
*Sapindus saponaria* L.	Sapindaceae	Ritha	Tree	Spring	Nutmeat	Nutmeat is eaten raw	No
*Betula alnoides* Buch. -Ham. ex D.Don	Betulaceae	Sayur	Tree	All seasons	Bark	Bark eaten raw as well as boiled in water as tea	No
*Oenanthe javanica* (Blume) DC.	Apiaceae	Seto Simsag	Herb	Spring to autumn	Whole plant	Whole plant used as vegetables	Yes
*Bombax ceiba* L.	Malvaceae	Simal	Tree	All seasons	Spines	Thick spines of young tree are consumed as betel with leaves of *Lantana camara*	No
*Nasturtium officinale* R.Br.	Brassicaceae	Sim sag	Herb	All seasons	Whole plant	Whole plant cooked as vegetables	Yes
*Urtica dioica* L.	Urticaceae	Sisnu	Herb	Summer to autumn	Leaves and shoot tips	Leaves and shoot tips are cooked as vegetable and broth	Yes
*Cymbidium erythraeum* Lindl.	Orchidaceae	Sunakhari	Herb	Summer	Flowers	Flowers cooked as vegetables	No
*Grewia vestita* Juss.	Tiliaceae	Syalphusro	Tree	Autumn	Fruits	Fruits eaten raw	No
*Bauhinia purpurea* L.	Fabaceae	Taaki	Tree	Summer	Flowers	Flowers cooked as vegetables	No
*Cinnamomum tamala* (Buch.-Ham.) T.Nees & Eberm.	Lauraceae	Tejpata	Tree	All seasons	Leaves	Leaves used as spice	No
*Cycas pectinata* Buch.-Ham.	Cycadaceae	Thakal	Tree	Spring	Mature fruit and pith	Mature fruit eaten raw; pith cooked as vegetables	No
*Schizostachyum dullooa* Nees.	Poaceae	Thokrey Baas	Perennial grass	Summer	Tender shoots	Tender shoots cooked as vegetables	No
*Aconogonon molle* (D. Don) H. Hara	Polygonaceae	Thotney	Shrub	Summer to autumn	Tender shoot	Tender shoots eaten as salad	Yes
*Zanthoxylum armatum* DC.	Rutaceae	Timur	Tree	Autumn	Fruits and leaves	Fruits and leaves used as spice	No
*Solanum torvum* Sw.	Solanaceae	Titay	Shrub	Summer	Fruits	Fruits cooked as vegetables	No
*Oroxylum indicum* (L.) Kurz	Bignoniaceae	Tohtalo	Tree	Spring	Flowers	Flowers cooked as vegetables	No
*Tropaeolum majus* L.	Tropaeolaceae	**1 Locally identified	Vine	Summer to autumn	Flowers, leaves, and tender shoots	Flowers, leaves, and tender shoots are cooked as vegetables	Yes
*Pandanus* sp.	Pandanaceae	**2 Locally identified	Tree	Autumn to winter	Ripen fruits and pith	Ripened fruits consumed raw and pith cooked as curry	Yes

*Yes (Sekar, 2012 [78]; Sekar et al., 2012 [78]; Hiremath at al. 2017 [67], Panda, 2014 [87])

**1 Locally identified as edible by the (Nepali) common name Tamarkay Phul (Nepali) was not certain. **2 Locally identified as edible by the (Nepali) common name Tari Kath (Nepali) was not certain.

**Table 2:** Summary data on the consumption, plant type and harvest season of wild edible plants reported in Samdrup Choeling, Bhutan.

Methods of consumption	% of spp. (number)	Parts consumed	% of spp. (number)	Plant type	% of spp. (number)	Harvest season	% of spp. (number)
Raw	39.2 (47)	Flower	10.8 (13)	Herb	25. 8 (31)	Spring	26.7 (32)
Cooked	46.7 (56)	Leaf	20.8 (25)	Shrub	10.8 (13)	Summer	26.7 (32)
Pickled	15.8 (19)	Shoot	28.3 (34)	Tree	45.0 (54)	Autumn	16.9 (20)
As tea	2.5 (3)	Fruit	43.3 (52)	Vine	13.3 (16)	Winter	18.3 (22)
Chewed	8.3 (10)	Seed	6.7 (8)	Aquatic	0.8 (1)	All seasons	14.2 (17)
Roasted	2.5 (3)	Root	0.8 (1)	Perennial grass	4.2 (5)	-	-
Spice	5 (6)	Whole plant	4.2 (5)	-	-	-	-
Other*	9.2 (11)	Other**	10.8 (13)	-	-	-	-

*Juice extraction, resin, and latex

**Bark and spines.

### Demographic profile of respondents

There were 76 farmers (>20 years of age) who took part in the voluntary questionnaire survey. In terms of gender representation, 55.3% were male and 44.7% female farmers. Seventy-five per cent of the interviewees were between 20 and 50 years of age while the remainder were > 50 years old. The plurality of the respondents was 36.8% illiterate; few had a diploma/degree or higher education; while the remaining fell into other categories (non-formal education—11.8%, primary school—13.2%, and high school—30.3%).

### Statistical findings

**Fig 2:**
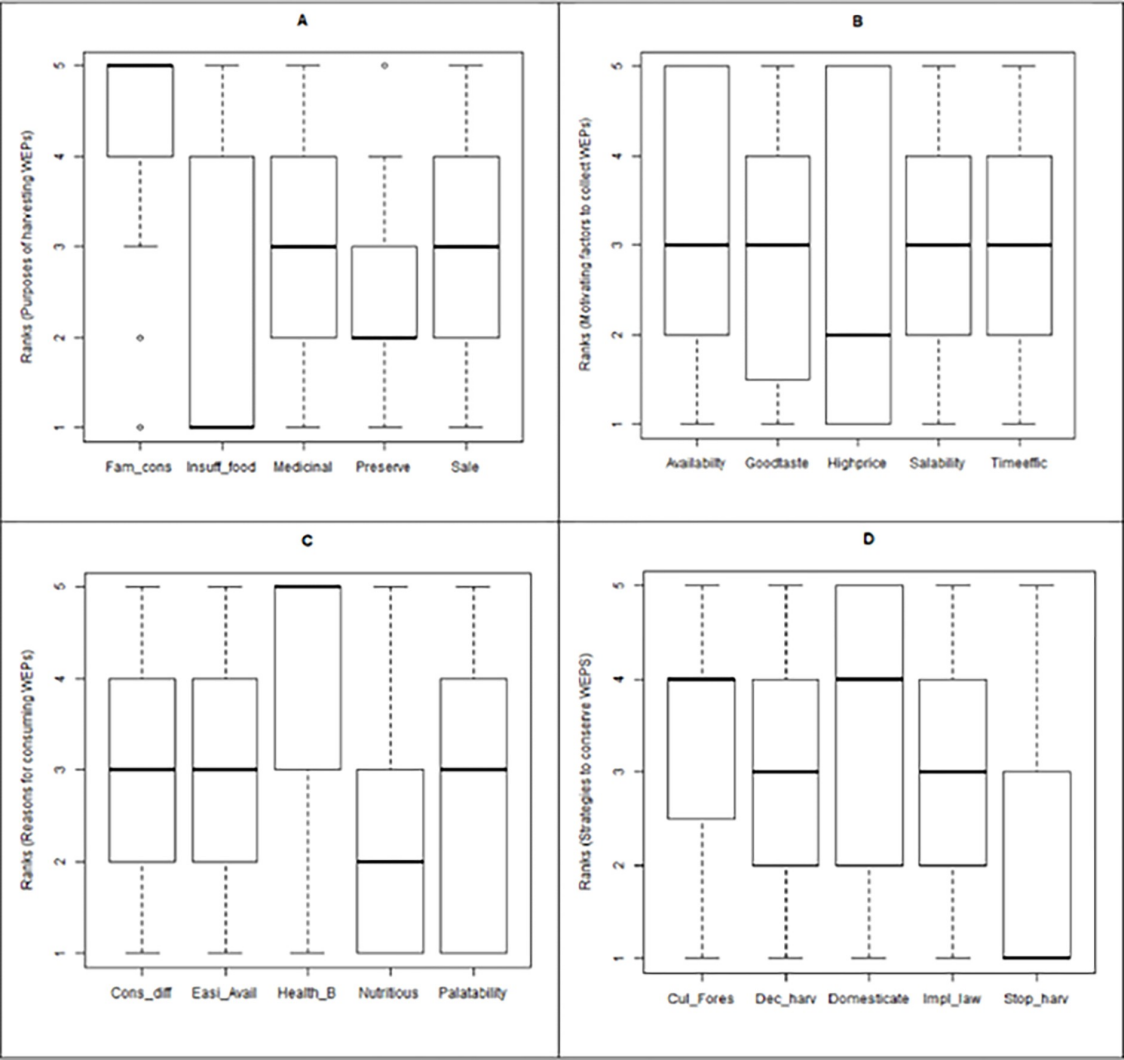
Local perceptions around the collection of wild edible plants (WEPs) reported by n = 76 informants in the Samdrup Choeling sub-district in Bhutan. In a two-stage process, a focus group identified four important aspects of WEP collection (A—purpose of harvest; B—motivation to harvest; C—reason for consumption; D—strategies for conservation). Informants then ranked each characteristic within the attribute. Data distribution by median, spread and skewedness of the four data sets is illustrated. Characteristics are abbreviated as: Fam_cons = family consumption, Insuff_food = insufficient food from cultivated sources, Medicinal = medicinal uses, Preserve = preserve for future use, Sale = for sale, Timeeffic = time efficient, Cons diff = consume in different ways, Easi_Avai = Easily Available, Health_B = health benefits, Cul_Fores = cultivate in forest, Dec_harv = decrease harvesting, Domesticate = cultivate in farm, Imple_law = Implement regulations and stop harv = stop harvesting.

**Fig 3:**
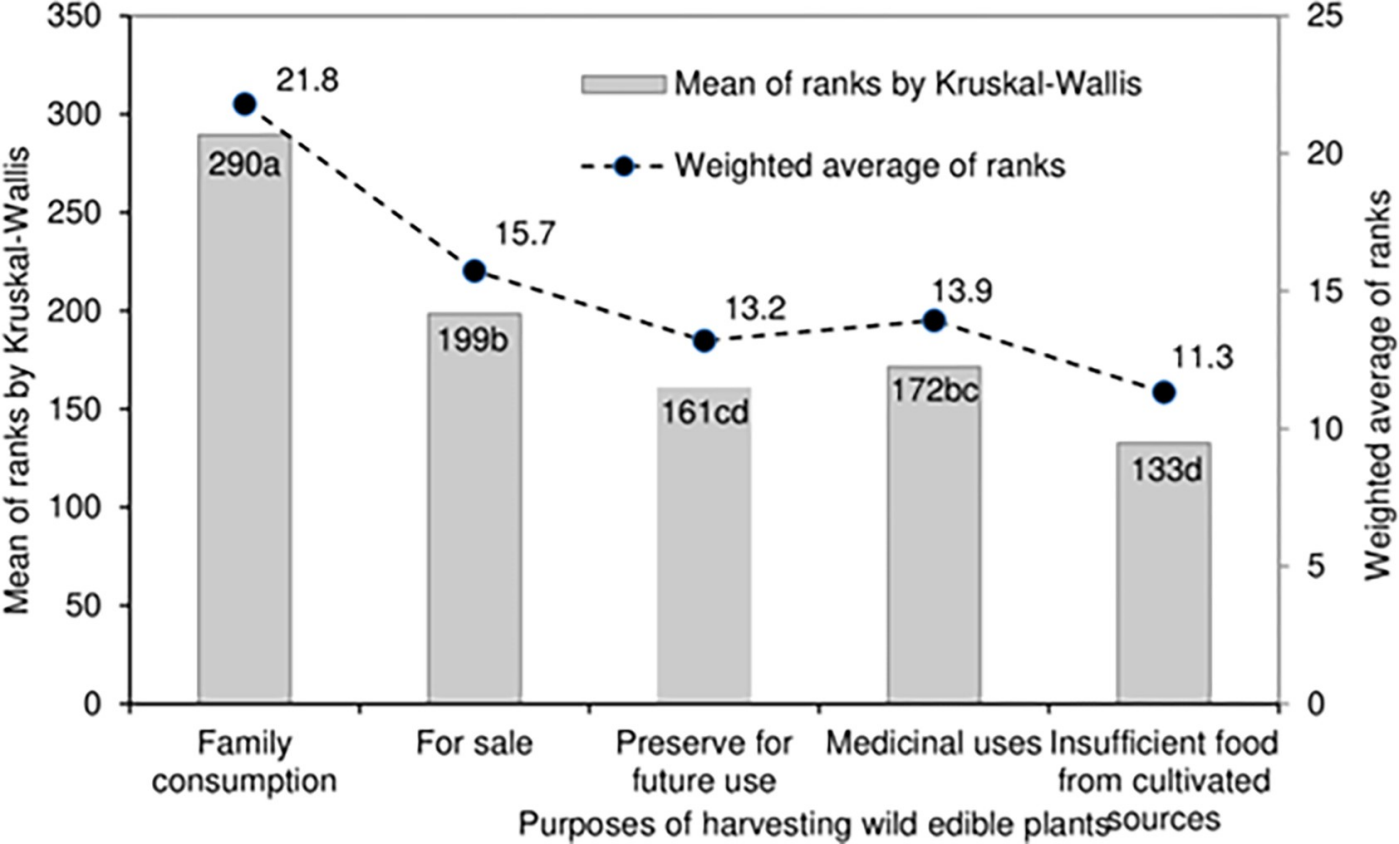
The five purposes of harvesting wild edible plants.

**Fig 4:**
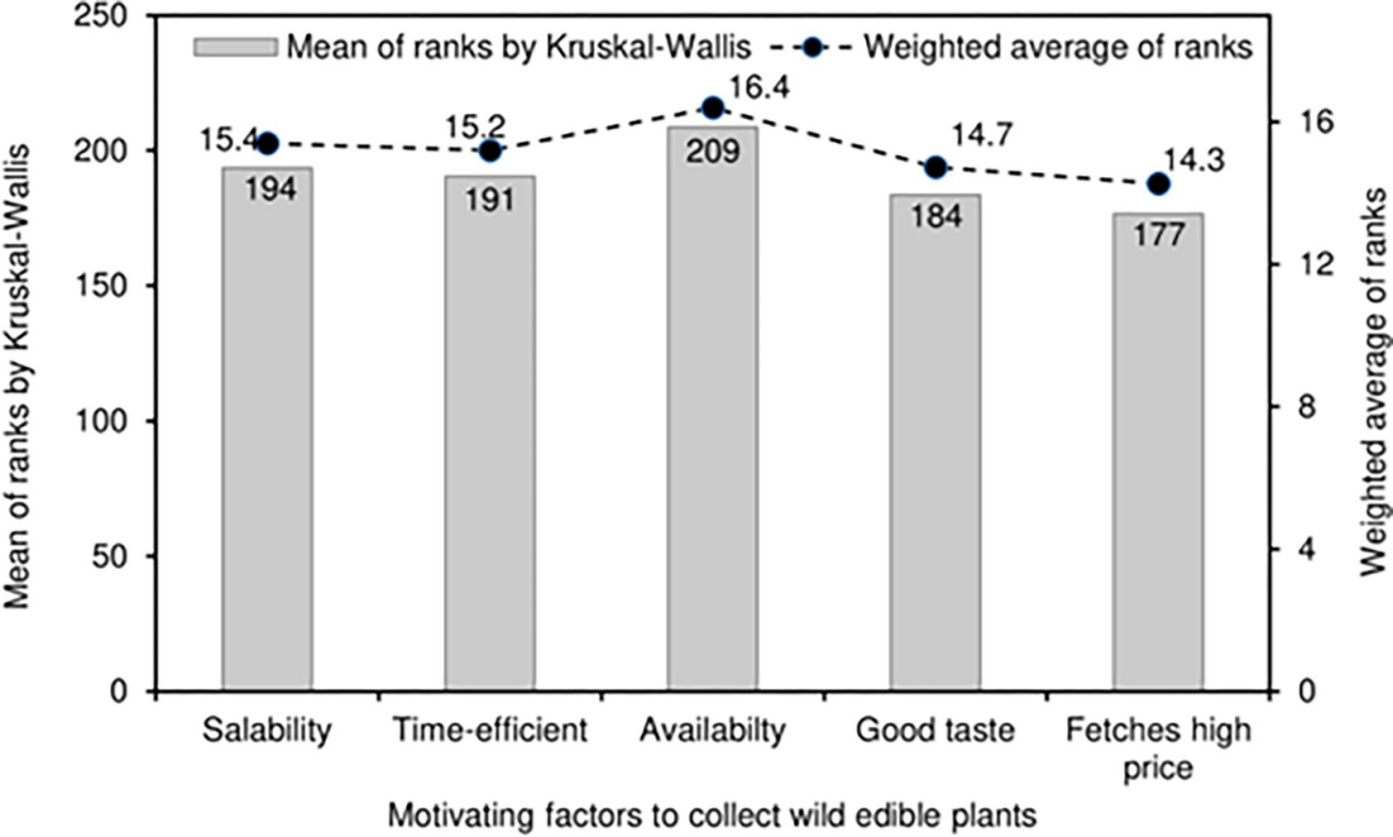
Motivating attributes to collect wild edible plants.

**Fig 5:**
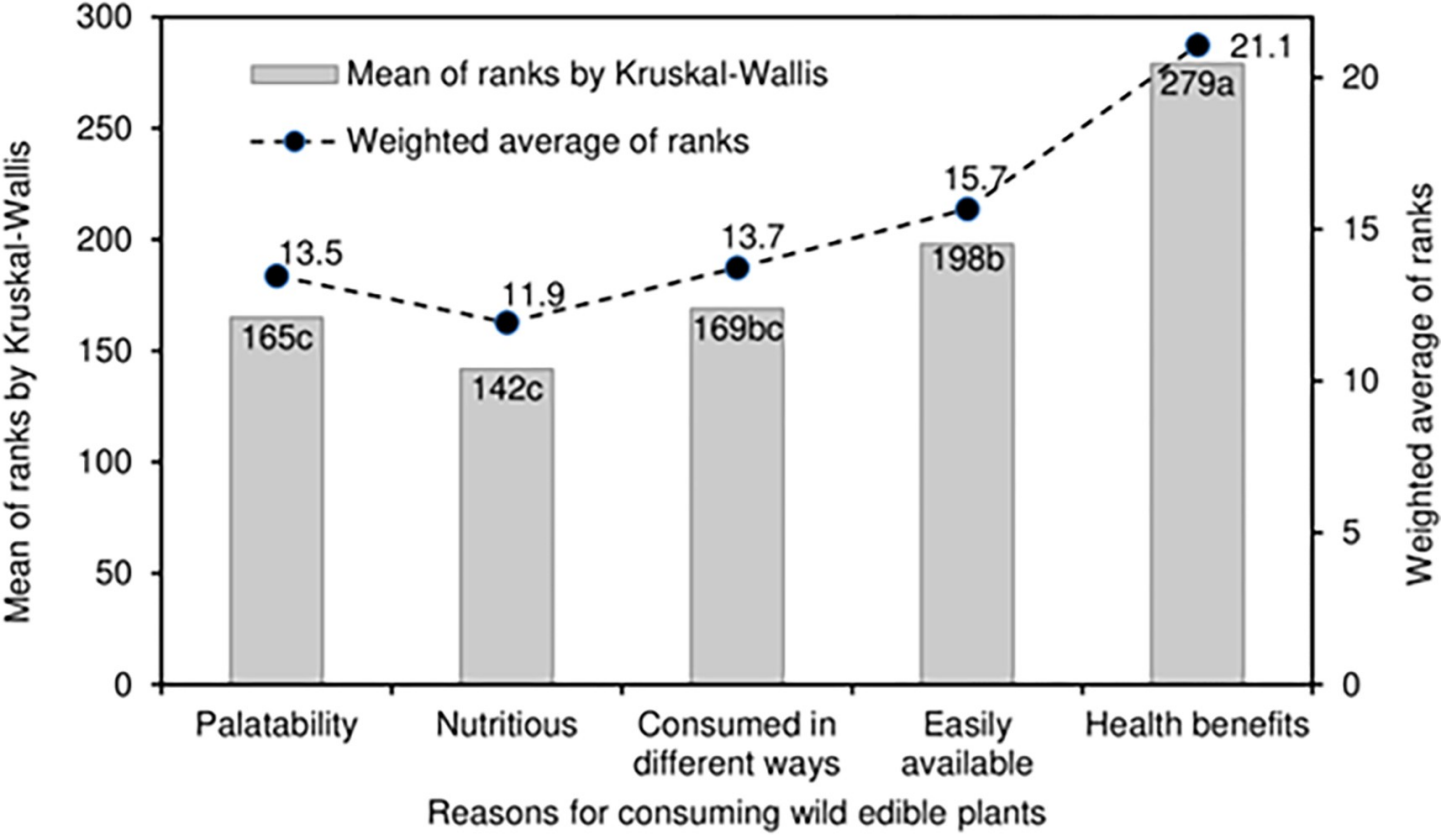
Reasons for consuming WEPs.

**Fig 6:**
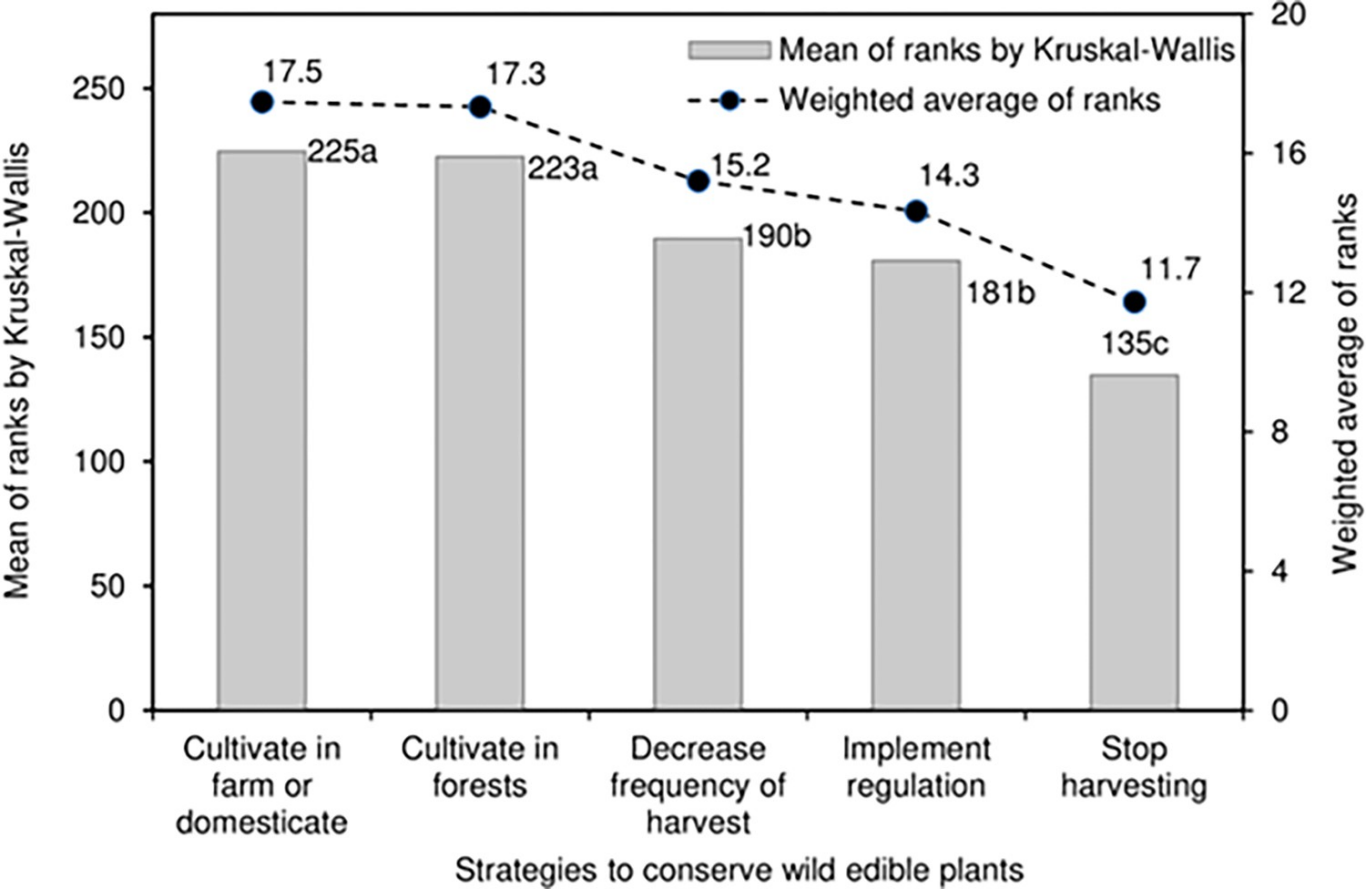
Strategies to conserve WEPs.

The median, spread and skewedness of the four data sets are graphically demonstrated (Fig 2) in boxplots to display the distribution based on 5 ranks used in the study (Fig 2). The non-parametric test of Kruskal-Wallis rank sum test for several dependent samples, which is equivalent to one-way ANOVA for parametric tests, was used to analyze the data were ranks or categorical. Reasons for harvest, consumption, and conservation of WEPs were highly and significantly different (P<0.001), while the motivating factors for collection were not (Table 3). Since the ranking scale used in the study was 5 = highest priority to 1 = lowest priority, a numerically higher mean rank implies a higher priority and vice-versa, therefore the mean ranks or the weighted averages should be elucidated in this context (Figs 3 to 6).

**Table 3:** Summary of Kruskal-Wallis rank sum test for several dependent samples. Kruskal-Wallis rank sum test showing consensus on local prioritization of purposes for harvesting wild edible plants (with family consumption highest, see Fig 3); reasons for consuming (with health benefits highest, see Fig 5); and strategies for conservation (with cultivation/domestication in farm / in forest highest, see Fig 6); but not for motivating attributes of collection (no significant difference, see Fig 4).

SN	Area of investigation/treatment	N	Df	Chi-square/test statistics	P-value
1	Purposes of harvesting WEPs	380	4	95.078	<0.001
2	Motivating factors to collect WEPs	380	4	3.7926	0.4348
3	Reasons for consuming WEPs	380	4	74.614	<0.001
4	Strategies for conservation of WEPs	380	4	35.426	<0.001

n = 380 because for each of the major aspects of WEPs had the five attributes ranked by 76 farmers (76 × 5 = 380).

### Purposes of harvesting wild edible plants

The data for medicinal uses and sale are symmetrically distributed, whilst that of preserve for future is skewed towards the lower quartile (39% respondents ranked 2^nd^ rank) (Fig 2A). However, data for family consumption and insufficient food are fully skewed in opposite directions. Family consumption is skewed towards upper extreme value (rank 5) as the median, lower quartile and upper extreme value coincided since 68% of the respondents ranked the purpose 5.

The results of Kruskal-Wallis rank sum test showed that most of the purposes of harvesting WEPs were highly and significantly different (P<0.001) to each other (Fig 3 and Table 3). The highest mean rank was for family consumption (290) since 68% of the respondents ranked it as 5 (Fig 2A), and this was significantly higher than the other four purposes of harvesting WEPs. The lowest rank was for the insufficient food from cultivated sources (133), a result of 53% of respondents categorizing this as rank 1 (Fig 2A): it was significantly lower compared with the other four purposes of harvesting WEPs, except for preserve for future use. Additionally, the data for insufficient food from cultivated sources is fully skewed towards the lower extreme value and the median coincides with a rank of 1.

The weighted averages values show the degree of importance and closely follow the mean ranks. The mean ranks and weighted averages for purposes of collecting WEPs were: family consumption > for sale > medicinal uses > preserve for future use > insufficient food from cultivated sources (Fig 3).

### Motivating attributes to collect wild edible plants

Data for good taste, salability and time-efficient are symmetrically distributed (rank 3 as median), while that of availability is slightly skewed towards the upper extreme value even though the median is rank 3 (Fig 2B). However, high price is skewed towards the lower extreme value with rank 2 as the median as 30% of respondents characterized it as rank 5. The statistical results of Kruskal-Wallis rank sum test showed that the motivating factors to collect WEPs were not significant (P = 0.435) to each other even though the mean ranks ranged from 177 to 209 (Fig 4 and Table 3). Moreover, the weighted averages ranged from 14.3 to 16.4 and closely followed the trend of mean ranks (Fig 4) hence, the null hypothesis of factors being the same is accepted.

### Reasons for consuming WEPs

The data for consume differently, easily available and palatability are fairly symmetrically distributed with a median rank of 3. However, for health benefits there is a strong skewedness towards the upper extreme value (median = rank 5) and 64% of the respondents ranked it as 5. The mean ranks for most of the reasons for consuming WEPs were statistically different (P<0.001) to each other (Fig 5 and Table 3) with health benefits (279) as the highest priority and nutritious (142) as the lowest. This is also indicated by the strong skewedness of data towards rank 5 for health benefits (Fig 2C) with 64% of the respondents, and the skewedness of nutritious to rank 1 with 36% of the respondents. The mean ranks of palatability, easily available, and consumed in different ways did not differ significantly from each other even though the mean ranks ranged from 165 to 198 (Fig 5). Additionally, the weighted averages which ranged from 11.9 to 21.1 closely followed the trend in mean ranks.

### Strategies to conserve WEPs

The data for decrease harvest and implement regulations are symmetrically distributed (median = rank 3), while those for cultivate in forest and domesticate are skewed towards rank 5 (median = rank 4) (Fig 2D). However, stop harvesting is fully skewed towards the extreme lower value (median = rank 1) because 51% of the respondents categorised it as rank 1. The Kruskal-Wallis rank sum test showed that the strategies to conserve WEPs were significantly different (Fig 6 and Table 3) at P<0.001. The two most important strategies to conserve WEPs were to cultivate in farm (or domesticate) and forests as these had significantly (P<0.001) higher mean ranks (≥223) compared with the other three strategies (≤190); this can be further verified by the graphical data distribution showing skewed data towards rank 5 (median = 4) (Fig 2D).

The least important strategy to conserve WEPs was to stop harvesting (mean rank = 135) and this had a significantly lower mean rank than the other four strategies (Fig 6). Strategies of decreasing frequency of harvest and to implement stringent regulation were of moderate importance and were significantly different to the other three strategies (Fig 2D). Further, the data for stop harvesting is strongly skewed towards rank 1 with 51% of the respondents categorising it as this. Further, the data trend is cross-validated using the weighted averages as they closely follow the mean ranks.

## Discussion

### Presence, consumption, plant types and seasonality of WEPs

Bhutan is one of the 10 global hotspots for biodiversity [81]. The large number of WEPs species (120 from 63 families) reported (Table 1) is reflective of this, due mainly to the favourable subtropical conditions suitable for a diverse range of plant species. A separate study reported 99 species in 55 plant families from Khoma village of the Lhuntse district in north east Bhutan [42]. Similar studies in neighbouring Bangladesh reported 102 WEPs [82], and in Nepal 85 WEPs [2]. The number of species reported can vary based of the geographical coverage as a major exploratory study in India reported observing 1403 species belonging to 183 families of WEPs [83]), in contrast to 41 species belonging to 17 families from the Manang district of Nepal [84]. A study in Sikkim found 190 species under 78 families [85] which is comparable with our findings.

Of 120 species consumed in our study, most were consumed cooked, raw, pickled, chewed or in other forms (Tables 1 and 2). Consumption method is dependent on the type of WEPs and difference are widely documented in literature from around the world [2,3,5,36,38,45,86,87]. Similarly, preparation methods depending WEP type including, eaten either cooked or raw, consumed as pickles, decocted as tea, chewed as betel, used for extracting the oil or juice, eaten as nuts by roasting, or even consumed as spices [2,3,5,36,38,45,50,86,87].

The most commonly consumed plant parts in the study were fruits (43.3%), shoots (28.3%) and leaves (20.8%) (Tables 1 and 2). This trend corroborates other studies in the region, for example in the northern Indian state of Uttarakhand [86] and in the Chinese region of Tibet [43]. Additionally, our findings match closely with the consumption preference from Bhutan and Nepal [42,88] and are comparable to a study from a reserve park in India [19], i.e., fruit > shoot > leaf.

The WEPs reported in the study were also classified into plant types (Table 1) with most of the WEPs recorded being trees (45.0%), herbs (25.8%), vines (13.3%), and shrubs (10.8%) (Table 2). Recent literature from neighbouring India supports our findings on the composition of plant type [86,87]. A major percentage of the WEPs species were harvested in spring and summer, possibly due to favourable seasonal plant growth, however only 14.2% were harvested year-round. Twenty-five per cent of WEPs reported were invasive which presents a concern for the long-term survival of native species [e.g. 89].

### Purposes of harvesting WEPs

The purposes for harvesting WEPs were significantly different (P<0.001) to each other (Fig 3) with the most important being for family consumption (68% of the respondents ranked it as 5 (Fig 2A)) and the least important being insufficient from the cultivated sources’ a trend also reported by others [19]. This is because WEPs are known to diversify the family food basket and meet the nutritional requirements of the local people [31].

The median of for sale and medicinal uses was rank 3 (Fig 2A) and they did not differ significantly from each other (Fig 3). This suggests the moderate role played by the WEPs for providing healthy food, and as a means to generate cash income. The lowest median and mean rank were for the insufficient food from cultivated sources indicating that farmers have the potential to be self-sufficient from their farming activities to some extent. In contrast, 64% of the respondents harvested wild *Dioscorea* species due to the insufficient rice and maize yields from cultivation in Nangkor in the Zhemgang district in central Bhutan in a separate study [28]. Further, Dema and Dolkar [42] reported that only 10% of WEP collections were for sale and that 90% were for the domestic consumption because access to markets was limited in that northern central area. The attribute of preserve for future use was also rated significantly lower possibly due to a lack the storage such as deep-freezing facilities or ignorance of modern processing and storage techniques. In other words, lesser quantities of WEP foods were likely to be preserved for consumption in lean seasons.

The relative importance of the purposes for collecting WEPs were family consumption > for sale > medicinal uses > preserve for future use > insufficient food from cultivated sources indicating varying degree of role played by the WEPs in supporting the rural livelihood in general. Moreover, the data of Kruskal-Wallis rank sum test closely match that of the weighted averages across all the analyses confirming the findings.

### Motivating attributes to collect WEPs

The common motivating attributes of local people collecting WEPs were assessed by ranking the five questions on salability in the market, time efficiency for collection, availability in the vicinity of the locality, they tasted good, and ability to generate high price. The median, which is a measure of central tendency for a non-parametric analysis, which is same for all five attributes (rank 3) (Fig 2B) except for fetches high price with the median of rank 2, implying a non-significant difference. Although the motivating attributes differed slightly numerically (177 to 209) in terms of by Kruskal-Wallis rank sum test, they did not differ significantly (P = 0.435) (Fig 4) indicating similar priorities or degrees of importance to the respondents. The weighted averages of the motivating attributes (range = 14.3 to 16.4) followed closely with the trend of mean ranks reinforcing the validity of the finding.

In contrast to this study, WEPs collected and sold in Nepal were reported to fetch high prices [2,23]: the differences could be related in contextual variation. In South Africa, the high price of WEPs has been a motivation for the poor households to harvest and sell WEP commodities in the local market [9]. Similarly, Namgyel [59] reported that the high price of *Cordyceps* (a genus of fungi widely used for medicinal purposes) as the motivation to harvest it from localites in the Bhutanese highlands.

### Reasons for consuming WEPs

The five most important reasons for consuming WEPs were palatability, nutritious, consumed in different ways, easily available and health benefits or of medicinal value. The mean ranks for the reasons for consuming WEPs were statistically different (Fig 5) with health benefits the highest priority and the nutritious as the lowest. There are important reasons why WEPs should be, and are consumed by the local people, these include the overlapping reasons of use as food and for medicinal value [87]. The reasons of palatability, nutritious and consumed in different ways did not differ (had the same median, Fig 2D), and it can be inferred that these were of similar priority to the respondents. Good taste, and freely available were the two most important perceptions while cultural identity was a negative indicator demonstrated by the indigenous communities in India [19]. Since WEPs grow naturally in the wild they are the equivalent of organically produced foods [50,90]), and are nutritious and palatable. Fungo [45] reported that consumption would increase four times if people knew that WEPs foods were more nutritious than foods from a cultivated source. In contrast, our research revealed that local people were aware of the palatable and nutritious values of the WEPs, and that health benefits featured very strongly with 64% of the respondents ranking it as rank of 5 (highest priority) (Fig 2D) even though a majority of farmers (61.8%) had ≤ primary school education.

### Strategies to conserve WEPs

An understanding of local people’s priority for WEPs conservation strategies were projected from their ranking responses because WEPs are under various constant natural and anthropological threats [43,88]. The identified strategies to conserve WEPs were significantly (P<0.001) different (Fig 6, Table 3), and the two most important strategies were to cultivate in farm or domesticate and cultivate in forests, both of which had a median score of rank 4 (Fig 2D). Thirteen species of WEPs are already domesticated by farmers in north-central Bhutanese district of Lhuentse in congruence to the findings of others [42]. According to Bharucha and Pretty [65] people who depend on forest resources maintain resources by the deliberate sowing of wild seeds, burning dried leftovers to stimulate plant growth, leaving portions of roots or replanting material, and extraction of only the required and useful parts while harvesting; these are synonymous to agricultural activities designed to enhance WEPs’ productivity. In northern Thailand, rice farmers plant many wild edible plants along irrigation canals, swamps, field boundaries and roadsides [91]. Many WEPs are also cultivated in the home gardens, which can become refuges for WEPs threatened by urbanization, drought, and deforestation [92]. WEPs such as *Artocarpus heterophyllus* Lamk., *Bauhinia purpurea* L., *Bauhinia retusa* Roxb., *Colocasia esculenta* (L.) Schott, *Emblica officinalis* Gaertn., were reported to be domesticated in home gardens in Odisha, in eastern India [87]. People who hold primary ownership of WEPs are key stakeholders of WEPs for conservation [59]. Hence conservation measures should also be aimed at these people and scaffolded to include their understanding of conservation issues.

Local people consider decrease frequency of harvest and implement of regulation as other prioritised methods (median = rank 3) of conserving WEPs because the high prices in the local markets could stimulate over-harvesting behaviour leading to conservation concerns such as those reported in Nepal [88]. Likewise, the decline of *Dioscorea* in Nangkor Geog under Zhemgang district in south central Bhutan [28] is clear evidence of the negative impact of over-harvesting. Similarly, in Cameroon, a WEP (*Gnetum africanum* and *G*. *buchholzianum*) has been pushed towards vulnerable status due to over exploitation [9]. Besides affecting conservation efforts, over exploitation will reduce the availability of WEPs in the long term and thus negatively impact the nutrition security of the local people [65]. The least prioritised conservation strategy was to stop harvesting because 51% of the respondents categorized it as rank 1 (median = rank 1). Further, a stop harvesting strategy may be perceived by farmers as a way of deprivation from WEPs collection, and thus negatively affecting their food and nutrition security. Hence, cultivating WEPs in farm and forest contexts are better conservation strategies based on the respondents’ opinions. In Bhutan, local people have noticed ecosystem changes and declining trends in the availability of forest resources [46]. In response, the government has developed a strategy for the sustainable harvest and conservation policy of non-wood forest products including the WEPs [60,93], which has had a tremendous impact on how we perceive and manage our forest resources [59], even though it may take long time to realise the expected impact.

Most studies have not considered the status on WEPs as native or invasive. In the current study, 25% of 120 species reported were invasive (Table 1), which potentially results in both negative and positive impacts [94,95]. Negative impacts include the ability of invasive species to suppress native species and threaten bio-diversity [e.g. 89,96], while positive impacts can include harvesting helping to curb invasiveness [2,3,86,97] so long as replanting and futher spread does not occur. *Lantana camara* L. is an example of invasive WEP reported in Samdrup Choeling [67,87], and previously planted as an ornamental across Bhutan [98]. Consuming invasive plants can help bridge food security and garner extra income for the rural people. There are many WEPs which are invasive and some examples are: *Tropaeolum majus* L., *Boerhavia diffusa* L., *Aconogonon molle* (D. Don) H. Hara, *Bidens pilosa* L., *Girardinia diversifolia* (Link) Friis, *Urtica dioica* L., *Nasturtium officinale* R.Br., *Oenanthe javanica* (Blume) DC., *Adhatoda vasica* L., *Houttuynia cordata* Thunb., *Solanum nigrum* L., *Amaranthus spinosus* L., *Pteridium aquilinum* (L.) Kuhn, *Remusatia vivipara* (Roxb.) Schott., *Elatostema lineolatum* J.R.Forst. & G.Forst., *Chenopodium album* L. and *Boehmeria* sp Jacq. [77,78,98]. Many of these species have not previously been recorded as present in Bhutan and need to be added to an updated National inventory of alien species [98]. The presence of these invasive species raises conservation concerns due their ability to suppress native WEPs and to cause a range of other negative impacts. Prompt risk assessment and prioritisation for management would be prudent [99].

## Conclusion

The presence of 120 WEPs species belonging to 63 families (including one Agaricaceae) were reported. Most of the WEPs recorded were trees followed by herbs, vines and shrubs, while the commonly consumed plant parts were fruit, shoots and leaves. The relative importance of the purposes of collecting WEPs were family consumption > for sale > medicinal uses > preserve for future use > insufficient food from cultivated sources. There are important reasons why WEPs should be and are consumed by the local people, including the overlapping reasons of food and medicinal value from WEPs. The data revealed that the palatability, nutritious and consumed in different ways categories of WEPs were of similar priority, whereas the health benefits associated with consumption was of the highest priority to the respondents. Two of the most important conservation strategies were to domesticate and cultivate in forests. The data of Kruskal-Wallis rank sum test closely match with that of weighted averages across all the analyses reinforcing the findings. The results reveal a range of valuable understandings and insights into the purposes of harvesting, motivations for collection, reasons for consumption and strategies on conservation of WEPs.

## Supporting information

S1 File (XLSX)
