# Correction: The presence of wild edible plants and determinants influencing their harvest, consumption, and conservation in south eastern Bhutan

**DOI:** 10.1371/journal.pone.0308232

**Published:** 2024-07-29

**Authors:** The PLOS ONE Staff

The affiliation 2 and 3 of the second author should have been captured as single affiliation not as different entities. Yadunath Bajgai is affiliated with: National Potato Program, National Centre for Organic Agriculture, Yusipang; Department of Agriculture, Ministry of Agriculture and Forests, Thimphu, Bhutan.

The ORCID iD is missing for the second author. Please see the author’s respective ORCID iD here:

- Author Yadunath Bajgai’s ORCID iD is: 0000-0001-9822-2581
  (https://orcid.org/0000-0001-9822-2581).

The publisher apologizes for the errors.
